# The impact of care process development and comorbidity on time to surgery, mortality rate and functional outcome for hip fracture patients: a retrospective analysis over 19 years with data from the Swedish National Registry for hip fracture patients, RIKSHÖFT

**DOI:** 10.1186/s12891-019-3007-0

**Published:** 2019-12-26

**Authors:** Emma Turesson, Kjell Ivarsson, Karl-Göran Thorngren, Ami Hommel

**Affiliations:** 1 Department of Orthopaedics, Skåne University Hospital, Lund, Sweden; 2 Department of Clinical Sciences Lund, Faculty of Medicine, Lund University, Lund, Sweden

**Keywords:** Hip fracture, Outcome, Care process development, Timing to surgery

## Abstract

**Abstract:**

For a long time the attention given to the hip fracture patient group was minor and without any certain consideration to their frailty. To improve the care for these patients Skane University Hospital in Lund has during the past 19 years worked actively with developing the care. This paper aims to describe what impact the care process development has had on functional outcome and mortality, as well as to analyze the impact of comorbidity and fracture type.

**Methods:**

Patients older than 50 years with non-pathological cervical and trochanteric hip fracture admitted between Jan 1st 1999 and Dec 31st 2017 were included and data was retrieved from the National Quality Register for hip fracture patients, RIKSHÖFT. Variables regarding patient characteristics, fracture type, operation method, lead-times and outcome were analyzed. For comparison Fischer’s exact test and Spearman’s rank correlation coefficient was used for the categorical data and Pearson correlation coefficient for the continuous. To further analyze the effect over time a linear regression model was used.

**Results:**

A total of 7827 patients were included. A significant shift in the overall morbidity was seen, with an increase in patients of higher ASA grade. No correlation was seen between outcome and the care process development. The mortality rate for the group as a whole the mortality rate had decreased over time. The total length of stay had decreased significantly over time. There was no statistically significant change in mortality rate over time when relating it to time-to-surgery.

**Conclusions:**

Although the patients display a higher morbidity over time, the mortality rate has not changed significantly, which might indicate an effect of the care process development.

The care process development does not seem to impact on outcome as much as other factors.

This study supports the possibility to create a more specific algorithm for hip fracture patients, taking specific subgroups into consideration.

## Background

To suffer from a hip fracture is a serious condition associated with high mortality, risk of complications and a decrease in functional level [1–5]. For a long time the attention given to this patient group was minor and the patients were attended to without any certain consideration to their frailty. Up until the 90’s the major focus in the orthopedic community was on developing the operation methods and for the treatment of hip fractures there has been a clear shift in regards to this. This shift, from osteosynthesis to hemiarthroplasty for cervical hip fractures and the introduction of intramedullary nailing for the trochanteric fractures, has been described in a previous study by the authors [6]. To improve the care for hip fracture patients Skane University Hospital in Lund has during the past 19 years worked actively with developing the care. In 1999 the first process development was made by changing the routines at the Emergency Department (ED) and taking action to prevent pressure ulcers. The changes included an active pain relief regimen, a nurse supervised waiting area at the ED and new pressure relieving mattresses in the wards and operating theaters [7]. Further improvements were made in October 2003 by introducing a new clinical pathway. With this clinical pathway the care of the patient started in the ambulance with distribution of pain relief, iv fluids and oxygen, and after x-ray the patient was taken directly to the orthopedic ward, as apposed to going back to the ED and wait for admittance. This led to a reduction of lead-times from X-ray to surgery [8]. The final step was made in April 2007 when the hospital implemented a fast-track care pathway to further reduce time from admission to surgery [9]. In this fast-track pathway the ED was completely bypassed and the patient was taken directly from the ambulance to X-ray and onwards to the orthopedic ward. The changes made led to a reduction in pressure ulcers and delirium, as well as a time gain from admission to x-ray and a reduced hospital stay.

The lead-time benefits with a fast track system have also been shown in other studies on hip fracture patients [10, 11].

The impact of time to surgery on the outcome after hip fracture has been the subject of many studies and debates during the past years and although all agree on the importance of not delaying surgery, the definition of “delay” varies, as shown in a paper by Lewis and Waddell from 2016 where they conducted an extensive review of the available literature [12]. The National Board of Health and Welfare in Sweden has set a goal regarding time to surgery that says that 80% of all hip fractures should be operated on within 24 h, in order to reduce complication rates and length of hospital stay, as well as to avoid rehabilitation delay [13].

The National Quality Register for Hip Fractures, RIKSHÖFT, has been used in Lund since the start in 1988 and has over the years had a high degree of coverage (85% nationwide in 2017 and > 95% in Lund) [14]. This gives us a unique opportunity to analyze the development in hip fracture care over a long period of time.

### Aim

The aim of this study is to investigate the impact of care process development and morbidity on time to surgery, mortality rate and functional outcome for hip fracture patients over a 19 year long period. No other studies have, to our knowledge, tried to describe the impact of care process development over such a long time period. A study like this can bring knowledge to the further development work for this important, and large, patient group.

## Patients and methods

Data from patients admitted for cervical or trochanteric fracture at Skane University Hospital in Lund from January 1st 1999 to December 31st 2017 were collected from RIKSHÖFT. Patients under 50 years of age and those with a pathological fracture were excluded. Data regarding general patient characteristics, fracture type, operation method, ASA (American Society of Anesthesiologists) physical status mental status, use of anticoagulants and functional parameters pre-fracture and at 4-months follow-up (housing and walking ability) was set up in a database together with information about date of admission, date of surgery and date of discharge. Since the register did not fully contain information regarding the specific time for admission/surgery/discharge, but just the dates, we registered the lead-times of interest (time-to-surgery and total length of stay (LOS)) in full calendar days, aware that there would be an overlap between the days. The days to surgery was defined as; 0 = operation within the same calendar day (date) as admission, 1 = operation within the first calendar day after admission (the next day), 2 = operation two calendar days after admission, ≥3 = operation three or more calendar days after admission.

As measures of morbidity we used the ASA classification system (see Table 2 for definition), cognitive status and use of anticoagulants. The information about ASA grade was included in the registry in 1998 whereas information on cognitive status and anticoagulant therapy were added to the registry in 2007. To assess functional level the variables ‘housing’ and ‘walking ability’ was used. These variables have several categories in RIKSHÖFT but for this study the categories were combined in order to make the analysis more clinically applicable. In Table 1 the recoding of the variables is presented.

**Table 1:** Recoding of variables

Variable	Old categories	New categories	Old categories combined
Admitted from/housing at 4 months	1. Own home	1.Own home	
	2.Group/service housing	2. Institutional care	2–6
	3. Full-service unit, nursing home		
	6. Rehabilitation unit, convalescent home		
	7. Acute hospital	3. Other	7–8
	8. Other		
Walking ability	1. Could walk alone outdoors	1. Independent walking ability	1–3
	2. Could walk accompanied outdoors		
	3. Could walk alone indoors		
	4. Could walk accompanied indoors	2. Dependent walking ability	
	5.Could not walk	3. Could not walk	

The table describes the recoding made for the different categories regarding housing and walking ability. The recoding was made to facilitate the analysis of the data and to make the results more clinically applicable

To enable an analysis of the impact of care process development the dataset was divided into three groups representing the three intervention periods described in the Introduction. The groups will onwards be referred to as TP (time period) 1 (January 1st 1999-Sept 30th 2003), TP 2 (Oct 1st 2003-Mar 31st 2007) and TP 3 (Apr 1st 2007-Dec 31st 2017).

### Statistics

To test the dependency and correlation between parameters we used Fischer’s exact test and Spearman’s rank correlation coefficient for the categorical data and Pearson correlation coefficient for the continuous. To further analyze the effect over time we used a linear regression model and applied a 95% confidence interval. For the regression model we also used R-square to determine the accuracy of the model. We set the statistical significance level to 0.05 for all analysis.

### Ethics

The study was approved by the Regional Ethical Review Board (ref.nr 2015/182). Upon registration in RIKSHÖFT the patients accept that their data may be used in research. No further approval from the patients has therefore been sought for this study. All data are presented on an aggregated level and the individual patient cannot be identified.

## Results

Between January 1st 1999 and December 31st 2017 a total of 7827 patients met the inclusion criteria set for this study, 71.1% were women. The mean age for the entire group was 81.9 (men 79.6, women 82.8). The men had significantly higher ASA grade (*p* < 0.001) than the women. For a more detailed description of the patients (both for the entire material and for each time period) see Table 2.

**Table 2:** Patient characteristics

	TP 1	TP 2	TP 3	All
n(%)	1760	1381	4686	7827
Mean age	81.7	81.5	82.1	81.9
Gender				
Female	1279(72.7)	1008(73.0)	3275(69.9)	5562(71.1)
Male	481(27.3)	373(27.0)	1411(30.1)	2265(28.9)
ASA grade				
ASA 1 (healthy)	122(6.9)	106(7.7)	196(4.2)	424(5.4)
ASA 2 (mild systemic disease)	906(51.5)	570(41.3)	1514(32.3)	2990(38.2)
ASA 3 (severe systemic disease)	637(36.2)	573(41.5)	2695(57.5)	3905(49.9)
ASA 4 (severe systemic disease with constant threat to life)	76(4.3)	95(6.9)	251(5.4)	422(5.4)
ASA 5 (moribund)	11(0.6)	3(0.2)	5(0.1)	19(0.2)
Admitted from				
Own home	1061(60.3)	904(65.5)	3315(70.7)	5280(67.5)
Institutional care	699(39.7)	476(34.5)	1247(26.6)	2422(30.9)
Other	0(0)	1(0.1)	124(2.7)	124(1,6)
Walking ability				
Independent	1604(91.1)	1297(93.9)	3770(80.5)	6671(85.2)
Dependent	52(3.0)	26(1.9)	784(16.7)	862((11.0)
Unable to walk	104(5.9)	56(4.2)	130(2.8)	290(3.7)
Fracture type				
Undisplaced cervical	272(15.5)	221(16.0)	610(13.0)	1103(14.1)
Displaced cervical	634(36.0)	519(37.6)	2092(44.6)	3245(41.5)
Two-fragment trochanteric	694(39.4)	567(41.1)	1023(21.8)	2284(29.2)
Multi-fragmentary trochanteric	160(9.1)	74(5.4)	961(20.5)	1195(15.3)
Operation method				
Two screws/pins/nails	381(21.7)	236(17.1)	753(16.1)	1370(17.5)
Sliding hip screw and plate	844(48.0)	622(45.0)	1723(36.8)	3189(40.7)
Intramedullary nail	2(0.1)	14(1.0)	254(5.4)	270(3.5)
Hemiarthroplasty	458(26.0)	447(32.4)	1526(32.6)	2431(31.1)
Total hip arthroplasty	53(3.0)	49(3.6)	382(8.2)	484(6.2)
Non-operative treatment	21(1.2)	12(0.9)	40(0.9)	73(0.9)

The table describes patient characteristics, both for the entire data set as well as for the different time periods representing the interventions made. TP 1 = Jan 1999-Sept 2003, TP 2 Oct 2003-Mar 2007, TP 3 = Apr 2007-Dec 2017)

**Fig. 1:**
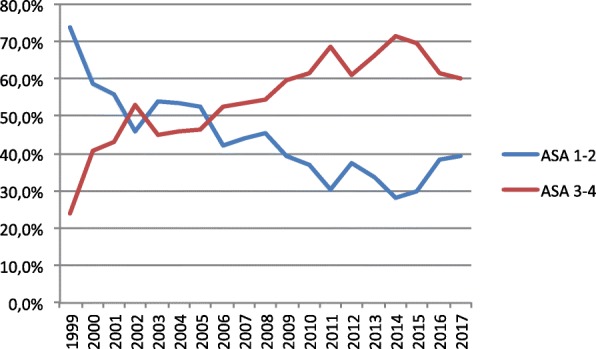
Morbidity changes over time. The figure shows the shift in morbidity over time

Over time there was a statistically significant shift (*p* < 0.001) in patient morbidity as shown in Fig. 1. The trend was the same for both men and women. We also see a statistically significant increase (*p* < 0.001) in use of anticoagulants from 6.6% in 2007 to 16.5% in 2017. The use of anticoagulants, as well as the mental status was not included in RIKSHÖFT until 2007 and for that reason no results prior to that are presented here. Both variables were statistically dependent on the ASA grade (*p* < 0.001).

Over time there is a decrease in mean age for patients with ASA 1 (75.4 years to 71.0 years) and ASA 2 (81.9 years to 80.3 years), whereas the mean age for ASA 3 and 4, with an overall mean age of 83.3 years and 82.6 years respectively, does not show any convincing time trend.

### Functional outcome, LOS and care process development

Our study shows statistic dependency between housing and walking ability both pre-fracture and at follow-up (*p* < 0.001), where independent walking ability correlates to living in own home. Over time there is an increase in number of patients living in own home pre-fracture (60.2% in 1999 and 75.1% in 2017) and at follow-up (51.8% in 1999 and 59.3% in 2017). When looking at walking ability we see a significant increase (*p* < 0.001) in dependent walking ability over time both pre-fracture (2.8% in 1999 and 29.2% in 2017) and at follow-up (8.0% in 1999 and 23.4% in 2017). The changes over time are not linear but happen from 1 year to another (2013 pre-fracture and 2006 at follow-up).

No conclusive time trend can be seen when relating ASA grade and functional outcome over time. When relating the functional outcome to the three interventions described in the introduction we see no clear relation.

Over the past 19 years we see a statistically significant decrease in LOS (*p* < 0.001) from 12.0 days in 1999 to 8.3 days in 2017. There was no statistically significant decrease in mean time to surgery (1.1 days in 1999 and 0.97 days in 2017, *p* = 0.11).

The LOS has changed with statistical significance within the first and last period (12.0 days in 1998 to 11.0 days in 2003 (*p* < 0.01) and 10.9 days in 2007 to 8.3 days in 2017 (*p* < 0.001)). No correlation was seen between the intervention periods and time to surgery.

### Mortality and time to surgery

The overall 4-months mortality rate for the entire group was 13,1% with a statistically significant decrease over time from 14.9% in 1999 to 10.0% in 2017 (*p* = 0.04). The mortality rate increased with age and ASA grade (Table 3). A statistically significant decrease in mortality is noticed both for ASA 1–2 (*p* < 0.001) and ASA 3–4 (*p* = 0.002) over the last 19 years.

**Table 3:** Mortality rate at 4-months follow-up

	TP 1	TP 2	TP 3	All	*P*-value
ASA					
1–2	9.0	8.1	5.4	7.0	< 0.001
3–4	22.4	18.4	16.2	17.6	< 0.001
Age group					
50–69	5.4	6.6	5.2	5.5	n.s
70–79	8.5	8.3	7.6	7.9	n.s
80–89	16.3	12.7	11.9	13.0	n.s
90+	24.2	27.1	22.0	23.3	n.s
Fracture type					
Undisplaced, cerv	12.9	10.0	11.0	11.2	n.s
Displaced, cerv	15.5	12.3	12.2	12.9	n.s
Two-fragment, troch	15.9	15.5	12.5	14.3	< 0.05
Multi-fragmentary, troch	11.3	20.3	12.9	13.1	< 0.05
Days to surgery					
0	14.8	15.5	11.2	13.0	n.s
1	13.8	11.6	12.6	12.7	n.s
2	13.2	14.2	11.6	12.5	n.s
3 or more	18.5	9.3	15.7	15.7	n.s

The table describes the mortality rate at 4-months for specific sub-groups as well as the relation between the three time periods (TP1 = Jan 1999-Sept 2003, TP 2 Oct 2003-Mar 2007, TP 3 = Apr 2007-Dec 2017). Days to surgery are defined as; 0 = operation within the same calendar day (date) as admission, 1 = operation within the first calendar day after admission (the next day), 2 = operation two calendar days after admission, ≥3 = operation three or more calendar days after admission. The *p*-value shows the statistical significant difference over time

In Table 3 the mortality rates for specific sub-groups are presented. For the fracture types we see the lowest mortality rate in the group with undisplaced cervical fractures and highest in the group with two-fragment trochanteric fractures. Over time, there is no change in mortality rate for the different fracture types, nor for the different age groups.

**Fig. 2:**
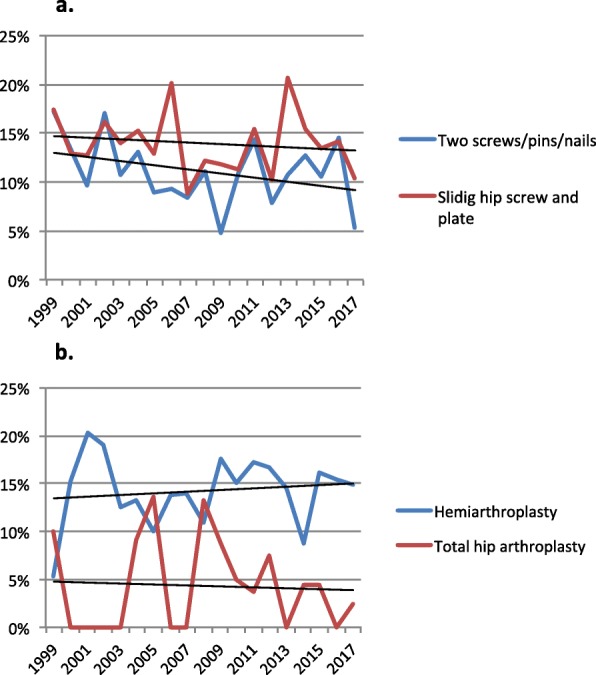
Mortality rate at 4 months and the change over time. The figure shows the mortality rate and the change over time for **a** the two most common methods for osteosynthesis in the material and for **b** hemi- and total hip arthroplasty

When looking at the different operation methods the results show a slight increase in mortality rate over time for those operated with hemiarthroplasty whereas osteosynthesis and total hip arthroplasty display a decreasing trend. The yearly variations are high which gives ambiguous results (Fig. 2a and b).

When correlating mortality and time to surgery the results show that those who waited three or more days from arrival to surgery had the highest mortality rate and those with shortest waiting time had the second highest. The difference between the highest and lowest mortality rate was 3.2% percentage points.

There was no statistically significant change in mortality rate over time for those operated within two calendar days from admission (in this paper defined as 0 and 1 day to surgery) although a decreasing time trend was seen. Neither could any difference be seen over time when looking specifically on those operated within the first calendar day.

**Fig. 3:**
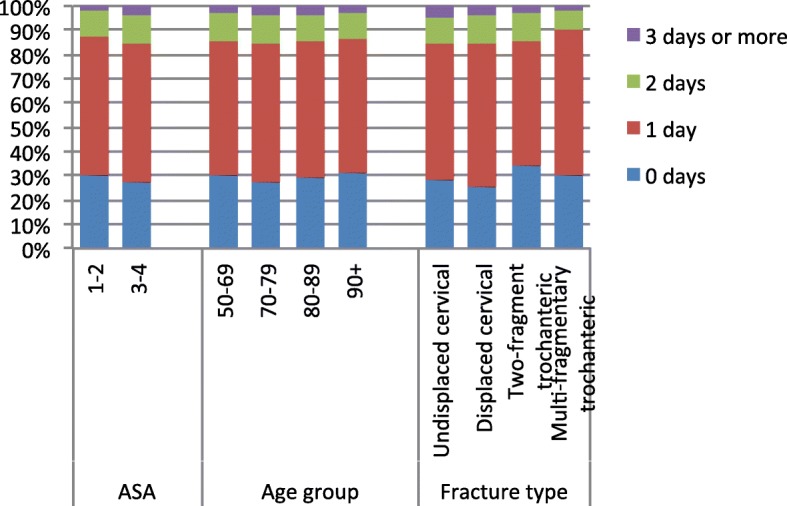
Time to surgery in relation to ASA grade, age and fracture type. The figure shows the waiting time to surgery, in calendar days, for different sub-groups and the distribution of the patients (in percent) between the four time groups. 0 = operation within the same calendar day (date) as admission, 1 = operation within the first calendar day after admission (the next day), 2 = operation two calendar days after admission, ≥3 = operation three or more calendar days after admission

When further studying time to surgery, as described in Fig. 3, we saw that most patients were operated within the first two calendar days from arrival (> 80%). We could see that patients in age group 70–79 were more likely to be operated two calendar days or later compared to the other age groups. We can also see this for those with ASA grade 3–4 and the patients with cervical fractures.

No difference was seen in regards to housing or walking ability when comparing those operated within the first or second calendar day, in both groups 71.3% of the patients had returned to own home at 4-months, whereas 61.2 and 62.9% had regained independent walking ability.

## Discussion

This study shows that the impact of care process development on time to surgery, functional outcome and mortality rate is limited but implies that other factors are of higher impact, such as patient morbidity, fracture type and age.

Our results support what previous studies have shown regarding age and gender distribution, showing that the women are both older and more numerous, even though the number of men sustaining a hip fracture have increased. Furthermore, the men are sicker and the trend is that the age when they sustain their fracture is increasing. Over time, the overall hip fracture patient population has become sicker and this could stand as one explanation to why the dependent walking ability has increased. But it could also be a result of misinterpreting the use of walking aids as a sign of decreased walking ability. In the beginning of the millennium the rollator increased in popularity and more patients were prescribed one upon discharge from the hospital. This could lead to the interpretation that the walking ability had declined even though that might not have been the case. To support this theory is the fact that more patients over time live in own home, both pre-fracture and at follow-up, suggesting a better overall function. To live in own home is however not a guarantee of a good functional level. In Sweden, the home care system has changed during the past decades with the result that fewer people are offered care in institutional living, even though they might be in need of that.

We have not been able to find any literature that describe the general development of walking aids in Sweden so our theories are based on own our clinical experiences.

Although the patients display a higher morbidity, increased age and use anticoagulant therapy in higher extent the time to surgery has remained steady around 1 day during the past 19 years. Also, the mortality rate has decreased over time for the group as a whole. This could be a result of all interventions made in the care for the hip fracture patients, but also a result of the general health care development. Even though it is hard from the results in our study to conclude any statistically significant effect on follow-up function and mortality in regards to the care process development, there might have been a different outcome if no changes in the management were made at all. It might be that the hip fracture patients are more fragile to begin with and therefor unable to make a better recovery, even with a thought-through management.

A limitation with this study is the use of the ASA-classification system as a measure of morbidity since this system is somewhat subjective and may also reflect the anaesthetists’ confidence and experience in managing this challenging patient group. However, to reduce this limitation the different ASA-classes have been combined into larger groups with the more healthy patients in one (ASA 1–2) and the sicker in one (ASA 3–4). This also makes the results more applicable in the clinical setting where patients are identified in a more general manner as healthy or sick.

In this study we could not see any impact on mortality rate in regards to time to surgery, even though the patients who waited three or more days had a higher mortality rate compared to those operated on sooner. This study did, however, identify subgroups with a higher mortality rate. This knowledge could help the orthopedic surgeon when planning the operation program, and act as guide when needing to prioritize between different hip fracture patients. But it also raises the question on care location. At our hospital, the hip fracture patients are cared for in an orthopedic ward, with orthopedic surgeons as responsible for the care. Hip fracture patients are however admitted to other wards as so called ‘outliers’ when the orthopedic ward is full. The patients are in those cases seen daily by an orthopedic consultant who is responsible for the care. In 2007 a decision was made to avoid placing hip fracture patients in other wards. This decision was based on a study by Hommel et al. showing increased LOS, delayed rehabilitation efforts and increased complications rates for hip fracture patients when treated in other hospital departments [15]. Our hospital has no tradition in orthogeriatric collaboration for this patient group, but maybe this ought to be the next step in the effort to improve outcome. Several studies have shown benefits in outcome when involving geriatricians in the care for hip fracture patients [16–18]. This study supports the possibility to create a more specific algorithm for hip fracture patients, including both the prioritization to surgery and care location.

When trying to assess care process development over 19 years one have to take into account all other changes that have occurred in the health care system and society. This makes it utterly hard to draw any conclusions without setting up a prospective randomized study. Also, it might be that the outcome measures chosen for this study are not the appropriate ones to study when evaluating this care process development. Other factors could be of greater importance, such as patient satisfactory and other care related factors. The real impact might be on a more subjective level and therefor harder to measure and follow with consistency.

In the RIKSHÖFT registry every hospital is able to set up own parameters for analysis. This has been made in the Lund registry after the implementation of the hip fracture care pathway in 2007 where data regarding patient inclusion in the care pathway is registered. To further attempt to answer the question regarding the impact of care process development we plan to conduct a more detailed study with this specific parameter in mind.

## Conclusions

Although the patients display a higher morbidity over time, the mortality rate has not changed significantly, which might indicate an effect of the care process development.

The care process development does not seem to impact on outcome as much as other factors.

This study supports the possibility to create a more specific algorithm for hip fracture patients, taking specific subgroups into consideration.

## Data Availability

The data that support the findings of this study are available from RIKSHÖFT but restrictions apply to the availability of these data, which were used under license for the current study, and so are not publicly available. Data are however available from the authors upon reasonable request and with permission of RIKSHÖFT.

## Abbreviations

ASA: American Society of Anesthesiologists
LOS: Total length of hospital stay

## References

[1] Foss NB, Kehlet H (2005). Mortality analysis in hip fracture patients: implications for design of future outcome trials. Br J Anaesth.

[2] Kannegaard PN, van der Mark S, Eiken P, Abrahamsen B (2010). Excess mortality in men compared with women following a hip fracture. National analysis of comedications, comorbidity and survival. Age Ageing.

[3] Rosell PA, Parker MJ (2003). Functional outcome after hip fracture. A 1-year prospective outcome study of 275 patients. Injury.

[4] Jacobs Hannes, Zeeb Hajo, Hoffmann Falk (2018). Incidence Rates of and Mortality after Hip Fracture among German Nursing Home Residents. International Journal of Environmental Research and Public Health.

[5] Sullivan KJ, Husak LE, Altebarmakian M, Brox WT (2016). Demographic factors in hip fracture incidence and mortality rates in California, 2000-2011. J Orthop Surg Res.

[6] Turesson E, Ivarsson K, Thorngren KG, Hommel A (2018). Hip fractures - treatment and functional outcome. The development over 25 years. Injury.

[7] Hommel A, Ulander K, Thorngren KG (2003). Improvements in pain relief, handling time and pressure ulcers through internal audits of hip fracture patients. Scand J Caring Sci.

[8] Hommel A, Bjorkelund KB, Thorngren KG, Ulander K (2007). A study of a pathway to reduce pressure ulcers for patients with a hip fracture. J Orthop Nurs.

[9] Turesson E, Ivarsson K, Ekelund U, Hommel A (2012). The implementation of a fast-track care pathway for hip fracture patients. Eur Orthop Traumatol.

[10] Larsson G, Stromberg RU, Rogmark C, Nilsdotter A (2016). Prehospital fast track care for patients with hip fracture: impact on time to surgery, hospital stay, post-operative complications and mortality a randomised, controlled trial. Injury.

[11] Hansson S, Rolfson O, Akesson K, Nemes S, Leonardsson O, Rogmark C (2015). Complications and patient-reported outcome after hip fracture. A consecutive annual cohort study of 664 patients. Injury.

[12] Lewis PM, Waddell JP (2016). When is the ideal time to operate on a patient with a fracture of the hip?: a review of the available literature. Bone Joint J.

[13] Öppna jämförelser av hälso- och sjukvårdens kvalitet och effektivitet. Stockholm: Swedish Association of Local Authorities and Regions; 2008.

[14] RIKSHÖFT annual report. Lund: Swedish National Registry of hip fracture patient care; 2017.

[15] Hommel A, Bjorkelund KB, Thorngren KG, Ulander K (2008). Differences in complications and length of stay between patients with a hip fracture treated in an orthopaedic department and patients treated in other hospital departments. J Orthop Nurs.

[16] Lau TW, Fang C, Leung F (2013). The effectiveness of a geriatric hip fracture clinical pathway in reducing hospital and rehabilitation length of stay and improving short-term mortality rates. Geriatr Orthop Surg Rehabil.

[17] Chuang CH, Pinkowsky GJ, Hollenbeak CS, Armstrong AD (2010). Medicine versus orthopaedic service for hospital management of hip fractures. Clin Orthop Relat Res.

[18] Forni S, Pieralli F, Sergi A, Lorini C, Bonaccorsi G, Vannucci A (2016). Mortality after hip fracture in the elderly: the role of a multidisciplinary approach and time to surgery in a retrospective observational study on 23,973 patients. Arch Gerontol Geriatr.

